# Predictors of Red Blood Cell Transfusion in Bimaxillary Orthognathic Surgery: A Retrospective Study

**DOI:** 10.7150/ijms.55567

**Published:** 2021-01-29

**Authors:** Seung-Hyun Rhee, Jung-Sub An, Kwang-Suk Seo, Myong-Hwan Karm

**Affiliations:** 1 Department of Dental Anesthesiology, Seoul National University Dental Hospital, Seoul, Republic of Korea.; 2 Department of Orthodontics, Seoul National University Dental Hospital, Seoul, Republic of Korea.; 3 Department of Dental Anesthesiology and Dental Research Institute, School of Dentistry, Seoul National University, Seoul, Republic of Korea.

**Keywords:** blood transfusion, hemoglobins, orthognathic surgery, red blood cell

## Abstract

**Background:** Orthognathic surgery requires red blood cell (RBC) transfusions more frequently than other oral and maxillofacial surgeries. The purpose of this study was to identify reliable predictors for RBC transfusion during bimaxillary orthognathic surgery (BOS).

**Methods:** This retrospective study reviewed 1,616 electronic medical records of patients who underwent BOS during a 5-year period at Seoul National University Dental Hospital. The perioperative variable data were collected from electronic medical records and analyzed by dividing patients into the two groups (non-transfusion and transfusion group).

**Results:** Of the 1,616 patients, 1,311 patients were excluded. The remaining 305 patients were divided into non-transfusion (NTF, n = 256) and transfusion (TF, n = 49) groups. Univariate logistic regression analysis revealed that age, body mass index, the presence of several adjunctive surgeries (including genioplasty, extraction, and mandibular angle reduction), preoperative hemoglobin (Hb) and prothrombin time, surgical time, amount of fluid infusion and blood loss, and mean pulse rate during surgery were significant factors predicting RBC transfusion. Multivariate logistic regression analysis revealed that preoperative Hb and blood loss amount during surgery were significantly related to RBC transfusion in BOS patients.

**Conclusion:** Since blood loss amounts could not be measured preoperatively, we found that the independent predictor associated with RBC transfusion during BOS was a low preoperative Hb level.

## Introduction

Red blood cell (RBC) transfusions are rare in oral and maxillofacial surgery, but in some cases of orthognathic surgery, it is required 1. The number of orthognathic surgeries that are performed each year is increasing due to their effectiveness in improving dentofacial anomalies. The possibility of blood loss during orthognathic surgery is high due to the poor visualization and high vascularity 2. Thus, orthognathic surgery has a higher frequency of RBC transfusion than other oral and maxillofacial surgeries 1, 3, 4. Several studies have shown that blood loss is greater during bimaxillary orthognathic surgery (BOS) compared to that of single-jaw orthognathic surgery 1, 5.

If there is a lot of bleeding during surgery, intraoperative RBC transfusion is inevitable, but it can cause several complications 6. RBC transfusions are associated with mortality and a significant increase in the length of postoperative hospital stay 4, 5. Several studies have introduced methods to reduce blood loss, including induced hypotensive anesthesia 7, 8, injections of tranexamic acid and desmopressin 9-11, and preoperative autologous blood donation (PABD) 12, 13 during orthognathic surgery. If the amount of blood loss can be predicted before BOS is performed, the surgical team can prepare for it and possibly prevent it. However, it is difficult to predict the amount of blood loss before BOS, as well as the potential for RBC transfusion. The operating time, segmentation of the maxilla, and preoperative values of the RBC count, hemoglobin (Hb), and hematocrit (Hct) can influence the amount of blood loss during orthognathic surgery 2, 14, 15. However, previous studies were conducted by various operators, and the reliability of the factors affecting blood loss conflicted between studies. In addition, few articles have been published on RBC transfusion-related factors in BOS.

To our knowledge, no article has demonstrated the reliable predictors of RBC transfusion during BOS. The purpose of the present study was to determine reliable predictors of RBC transfusion during BOS.

## Methods

This retrospective study was conducted at the Department of Dental Anesthesia at the Seoul National University Dental Hospital, Seoul, Republic of Korea. The need to obtain informed consent was waived since the data for the study were obtained solely from electronic medical records (EMRs) were reviewed. This single-center retrospective study was approved by the Institutional Review Board of the Seoul National University School of Dentistry (Approval number, S-D20190029). Under the approval of the Institutional Review Board, we collected EMR data from July 2014 to December 2019.

### Patients

The EMRs of 1,616 patients who underwent elective orthognathic surgery at our institute from July 2014 to December 2019 were reviewed for this study. The patients were diagnosed with mandibular prognathism, mandibular retrognathism, facial asymmetry, malocclusion, and obstructive sleep apnea. The inclusion criteria for this study included patients who underwent elective orthognathic surgery at our institute. Exclusion criteria were as follows: (1) patients who were not treated by a single surgical surgeon and single anesthesiologist; (2) patients who underwent re-fixation; (3) patients with the American Society of Anesthesiologists (ASA) patient status III or higher; (4) patients who underwent single jaw surgery; (5) patients who did not follow the pathway of normal orthognathic surgery, (e.g., total temporomandibular joint reconstruction, distraction osteogenesis, hemifacial microsomia); (6) patients who underwent PABD; (7) patients with coagulation disorders; and (8) patients with insufficient data in their EMRs.

### Anesthetic technique

All surgeries at our institute were performed by the oral and maxillofacial surgeon with more than 10 years of BOS experience and the anesthesiologist using the same protocol for each case. Standard preoperative evaluations, such as medical history, vital signs, routine laboratory tests, electrocardiography (ECG), and chest X-rays were performed for all patients within three months of surgery. No patients were given any preanesthetic medications before arriving in the operating room. Anesthesia was induced after establishing routine patient monitoring (pulse oximetry, ECG, and non-invasive blood pressure), bispectral (BIS) index monitoring, and sufficient preoxygenation. Two methods of anesthesia were performed: volatile and total intravenous anesthesia. For volatile anesthesia, anesthesia was induced with 1% propofol (1.5-2.5 mg/kg) or thiopental (4-5 mg/kg). When 1% propofol was used, 30 mg lidocaine was used prior to the propofol injection. For total intravenous anesthesia, the first target effect site concentration of propofol was set to 5 μg/mL with 5 ng/mL of remifentanil, which were administered with an Orchestra Base Primea target-controlled infusion system (Fresenius Kabi, Bad Homburg, Germany). For the neuromuscular blockades, a 0.5-0.8 mg/kg bolus of rocuronium was administered following anesthesia induction, and additional doses were administered as needed. After sufficient time for muscle relaxation, nasotracheal intubation was performed and mechanical ventilation was maintained in a volume control mode with 50% oxygen and 6-8 mL/kg of tidal volume with positive end expiratory pressure utilized as needed. The respiratory rate was adjusted to maintain end-tidal carbon dioxide partial pressure between 30 and 35 mmHg. Invasive blood pressure monitoring and arterial blood gas analysis (ABGA) were performed by placing a 20-gauge catheter in the dorsalis pedis artery. For volatile anesthesia, desflurane or sevoflurane was administered, and the minimum alveolar concentration was maintained between 0.9 and 1.2 depending on the BIS index level (40-60) and vital signs. For total intravenous anesthesia, propofol (2-4 μg/mL) and remifentanil (3-10 ng/mL) were used depending on the BIS index level (40-60) and vital signs. Several methods were used to reduce blood loss and the possibility of an RBC transfusion. Induced hypotensive anesthesia (mean arterial pressure 50-65 mmHg) was administered for procedures in which considerable blood loss was expected like down fractures of the maxilla, mandibular osteotomies, genioplasties, or mucosal incisions 16, 17. To check the patient's status, ABGA was performed every two hours during surgery. Urine output was measured every hour after bladder catheterization. Upon completion of the operation, residual neuromuscular paralysis was reversed using sugammadex. After recovery of self-respiration, the patients were transferred to a postanesthetic care unit. After confirmation of the complete recovery of consciousness, self-respiration (with sustained spontaneous respiration rate > 12/min), and an open airway, the tracheal tube was removed.

### Surgical technique: bimaxillary orthognathic surgery; Le Fort I osteotomy and bilateral sagittal split osteotomy

Before surgical incision, all patients were infiltrated with four to five dental lidocaine cartridges (2% lidocaine with 1:100,000 epinephrine) in the mucosa of the incision site. The procedure was initiated with a Le Fort I (LFI) osteotomy. After mobilization of the maxilla, the greater palatine canal and the descending palatine neurovascular bundle were identified. A large amount of bleeding was expected during the posterior procedure of the maxilla because of its rich pterygomaxillary vascular network 18. A judicious fracture of the pterygoid plates was performed to complete the osteotomy. The maxilla was subsequently moved to the planned position and any bony interferences were removed during this process. Mandibular surgery was performed using the bilateral sagittal split osteotomy (BSSO) technique 19. The technique used in our study involved splitting the mandible at the inferior border, which provides controlled positioning of the proximal segment.

### Transfusion criteria and grouping

The perioperative RBC transfusion approach was in accordance with recent standard transfusion guidelines 20-22. Indications for RBC transfusion included an Hb value below 7-8 g/dL and/or when an anesthesiologist determined that RBC transfusion was necessary. A delay in RBC transfusion until the end of the main procedure was attempted while maintaining the blood pressure with an infusion of crystalloids or colloids. All the data from the two patient groups were divided to assess the predictors of RBC transfusion: TF group (patients who were transfused with RBC) and NTF group (patients who were not transfused with RBC). In addition, the TF group was divided into two subgroups: a smaller volume transfusion group (same or less than two RBC packs) and a larger volume transfusion group (more than two RBC packs).

### Data collection

Data on potential factors correlated to intraoperative blood loss were retrospectively collected from the EMRs of patients who received elective BOS. Demographic data (age, gender, body weight, height, body mass index [BMI], preoperative vital signs, past medical and surgical history, and ASA classification) were collected. Laboratory data (including Hb, Hct, platelet count, activated partial thromboplastin time, international normalized ratio [INR], prothrombin time [PT], calcium, phosphorus, glucose, blood urea nitrogen, uric acid, cholesterol, total protein, albumin, total bilirubin, alkaline phosphatase, aspartate aminotransferase, alanine aminotransferase, creatinine, sodium, potassium, and chloride), chest X-rays, and 12-lead ECGs were collected. Intraoperative data (durations of surgery and anesthesia, amounts of drugs and fluid, estimated intraoperative blood loss [EBL], urine output, number of packed RBC transfusions, and ABGA data every two hours) were collected. Throughout the surgery, the mean value of oxygen saturation, pulse rate, end-tidal carbon dioxide, body temperature, systolic, diastolic, and mean arterial blood pressure were collected every 5 minutes. In addition, the mean value of pulmonary compliance, peak inspiratory pressure, and BIS were collected every 15 minutes. The EBL was calculated by subtracting the volume of saline solution used for irrigation from the total volume accumulated in the suction unit 23, 24. The blood in the gauze was not included in the EBL measurement.

### Statistical analysis

Baseline characteristics and pre- and intraoperative variables of patients who underwent BOS were compared to assess the differences between the TF and NTF groups. Continuous data were analyzed using the independent t-test and categorical data were compared with a chi-square test or Fisher's exact test.

The variables associated with RBC transfusions were included in the univariate logistic regression analysis. The multivariate logistic regression analysis was performed on selected variables (age, BMI, preoperative Hb, preoperative PT, and EBL) to evaluate the independent factors associated with RBC transfusions. The variables were selected based on biological plausibility, clinical significance, and statistical considerations. The goodness of the fit of the model was evaluated using the Hosmer-Lemeshow test.

Differences in the baseline and the pre- and intraoperative variables between the smaller (less than two RBC packs) and larger (more than two RBC packs) volume transfusion groups were analyzed using the Mann-Whitney U test. For all statistical analysis, IBS SPSS Statistics 25 was used and *P* < 0.05 was considered to be statistically significant.

## Results

**Figure 1:**
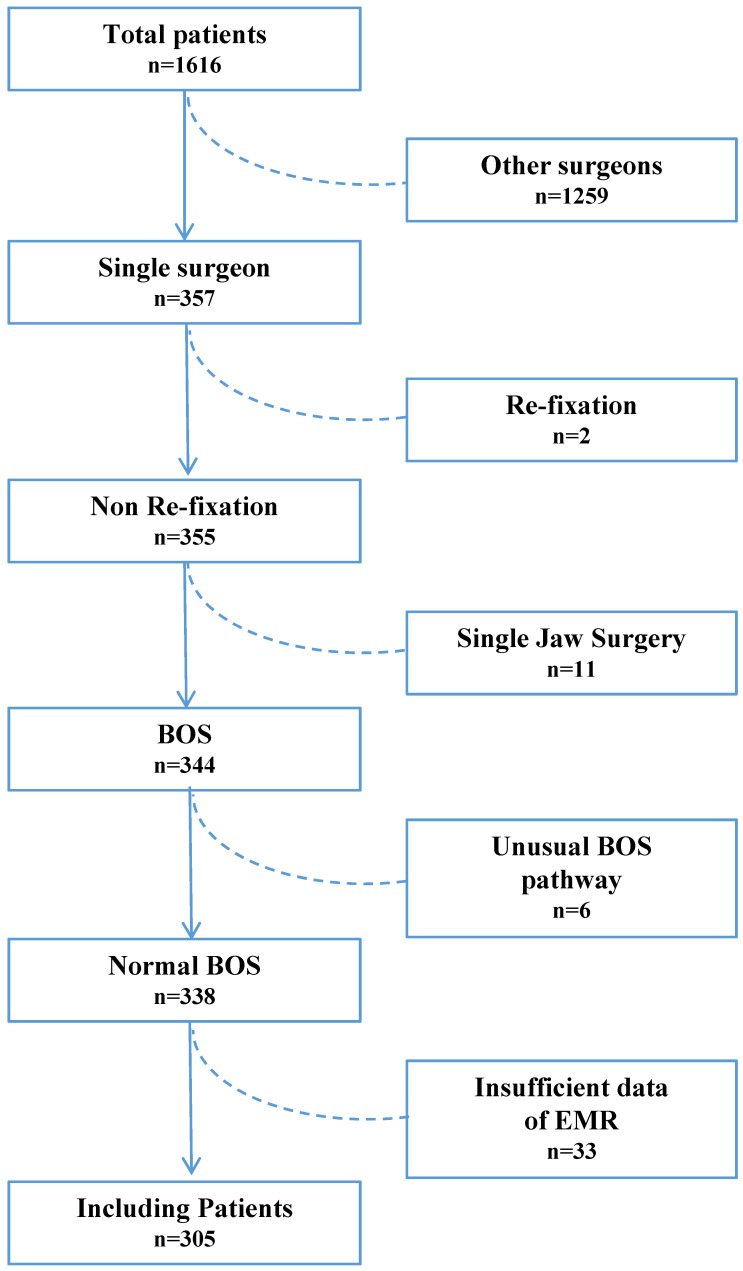
A flow diagram of the study population. BOS: bimaxillary orthognathic surgery; EMR: electronic medical records.

For eligibility, 1,616 patients who were scheduled to undergo orthognathic surgery between July 2014 and December 2019 at our institute were screened. Of these, 1,311 were excluded for the following reasons: 1,259 patients did not underwent a procedure completed by a single surgeon; 2 patients required re-fixation; 11 patients underwent single jaw surgery; 6 patients followed a unusual orthognathic surgery pathway like distraction osteogenesis (2 patients), total temporomandibular joint reconstruction (2 patients), iliac bone graft to hemifacial microsomia patients (2 patients); and 33 patients had insufficient data in their EMRs. Thus, 305 patients were selected for the study and were subsequently divided into the NTF (256 patients) and TF (49 patients) groups (Figure 1).

Statistically significant differences were found between the NTF group and TF group with regard to the baseline demographic data. Patients in the TF group were younger (*P* = 0.042) with smaller weight and height values (*P* < 0.001) and, thus lower BMI (*P* = 0.007) than the noes in the NTF group. There was no difference between the two groups in gender ratio, past medical and surgical history, and ASA classification (Table 1).

Some preoperative conditions differed between the two groups. In the TF group, body temperature was significantly higher (*P* = 0.011) while Hb and Hct were significantly lower (*P* < 0.001). Patients who received RBC transfusions (TF group) also exhibited higher INR and PT (*P* = 0.004). Other preoperative conditions were not significantly different between the two groups (Table 2).

In this study, three types of osteotomies were performed for the maxilla (LFI osteotomy, LFI osteotomy with anterior segmentation, and LFI osteotomy with Y segmentation) and two types of osteotomies performed for the mandible (BSSO, and BSSO with anterior segmentation). Additional procedures including genioplasties, segmental osteotomies of the maxilla, turbinectomies, tooth extractions, mandibular angle reduction, glossectomies, iliac bone grafts, and face lifts with threads were performed if necessary. The effects of the procedures on EBL and RBC transfusions were also investigated. Patients in the TF group received significantly more adjunctive surgical procedures than the NTF group including genioplasties (*P* = 0.007), tooth extractions (*P* = 0.006), and mandibular angle reduction (*P* = 0.001). The type of anesthesia used was not correlated with a statistically significant difference between the two groups (Table 3).

The TF group underwent longer surgery and anesthesia times than the NTF group and had more EBL, which resulted in higher amounts of crystalloid and colloid infusions (*P* < 0.001). In addition, the TF group exhibited a lower BIS index (BIS) (*P* = 0.023), higher mean oxygen saturation (*P* = 0.005), and faster mean pulse rate (*P* = 0.001) during surgery (Table 4).

ABGA was performed up to 5 times administered in 2-hour intervals. All patients (n = 305) underwent at least one ABGA. The 2^nd^, 3^rd^, 4^th^, and 5^th^ examinations were performed on 302, 269, 122, and 38 patients, respectively. Hb and Hct were significantly different between the two groups for all five ABGA results (*P* < 0.05). Partial pressure of oxygen was significantly different four times (1^st^, 2^nd^, 3^rd^, and 4^th^) (*P* < 0.05); peripheral oxygen saturation was different four times (1^st^, 2^nd^, 3^rd^ and 5^th^) (*P* < 0.05); and the calcium level was different three times (2^nd^, 4^th^, 5^th^) during each examination (*P* < 0.05) (Table 5).

**Figure 2:**
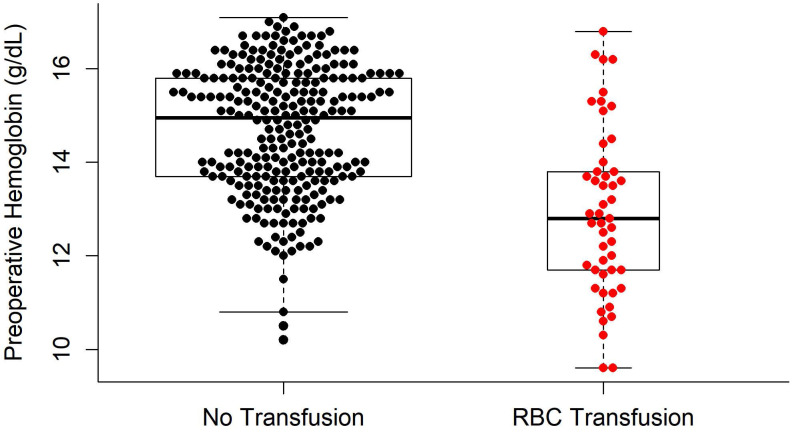
Comparison of preoperative hemoglobin levels between the RBC transfusion group and no RBC transfusion group. RBC: red blood cells.

Univariate logistic regression analysis revealed that age, BMI, presence of several adjunctive surgeries (including genioplasty, extraction, and mandibular angle reduction), preoperative Hb and PT, surgical time, amount of fluid infusion, EBL, and mean pulse rate during surgery were significant factors predicting RBC transfusions (*P* < 0.05; Table 6). In the multivariate logistic regression analysis, the independent variables that were significantly related to RBC transfusions in BOS were preoperative Hb (odd ratio [OR] = 0.413; *P* < 0.001) and EBL (OR = 1.005; *P* < 0.001) (Table 6). The difference in the preoperative Hb levels between the TF and NTF groups is shown in Figure 2.

Of the 49 patients who received RBC transfusions, five patients received one pack of RBCs; 34 patients received two packs; five patients received three packs; three patients received four packs; one patient received five packs; and one patient received seven packs. There was no significant difference in the variables between the smaller volume transfusion group (same or less than two RBC packs, n = 39) and the larger volume transfusion group (more than two RBC packs, n = 10) except for mean body temperature during surgery. The intraoperative mean body temperature of the larger volume transfusion group (35.3 °C) was significantly lower than that of the smaller volume transfusion group (36.0 °C) (*P* = 0.003) (Supplementary Table 1).

## Discussion

In the present study, we evaluated predictors of RBC transfusions in 305 patients who underwent BOS. According to the results of this study, patients of the TF group were younger and smaller (height, body weight, and BMI); exhibited lower preoperative Hb and Hct; and had higher preoperative INR, PT, and body temperature. The TF group had a longer surgery time than the NTF group, and accordingly was anesthetized for a longer period. As a result, the amount of fluid infusion was higher due to an increase in EBL. This was probably due to the fact that the TF group underwent adjunctive surgeries. In the TF group, the mean value of intraoperative oxygen saturation and pulse rate were higher and the BIS was lower than NTF group. The only significant difference among the intraoperative ABGA was Hb through 5 times of examinations. In the univariate logistic regression, preoperative Hb and PT, total fluid, EBL, and the mean value of the pulse rate exhibited significant differences among the groups. Among them, preoperative Hb and EBL were considered to be important factors affecting RBC transfusions in multivariate analysis. Since EBL is dependent on factors that are affected by surgery, preoperative Hb was the only factor that can predict RBC transfusion before BOS.

The TF group exhibited higher preoperative body temperature than the NTF group. However, in the TF group, the larger volume transfusion group (more than two RBC packs, n = 10) had lower perioperative body temperature compared to the smaller volume transfusion group (same or less than two RBC packs, n = 39). This result is probably due to the low temperature of the cold pack used for RBC transfusions. However, since both groups showed perioperative body temperatures within the normal range, this result is not considered to have significant clinical importance.

Surgical bleeding or PABD may affect RBC transfusion during BOS. Depending on the surgeon, the amount of bleeding may differ even during similar types of surgery. Therefore, to exclude differences between surgeons, 305 patients who underwent a procedure that was performed by the single surgeon were included in this study. PABD may affect the preoperative condition of the patient, so patients who performed PABD were also excluded.

In orthognathic surgery, RBC transfusions are relatively infrequent with a reported incidence rate of 2-19.5% 5, 23, 25. In the present study, 49 (16.3%) patients received RBC transfusions. The reason why our study exhibited a relatively higher incidence of RBC transfusions compared to other studies is that the need for transfusion is more conservatively determined than the current guidelines. Our indications for RBC transfusions included intraoperative Hb values lower than 7-8 g/dL and/or when an anesthesiologist determines that RBC transfusion is necessary. Factors that the anesthesiologist in this study considered when deciding to initiate a RBC transfusion include sudden and rapid bleeding, such as arterial bleeding, large blood loss relative to body weight, and unfavorable vital signs.

It has been reported in previous studies that EBL, operative time 15, 23, adjunctive surgical procedures 26, 27, gender, and experience of the surgeon 28 are factors that influence RBC transfusion during orthognathic surgeries. In the present study, the operating time and EBL also exhibited significant differences between the TF and NTF groups. However, these factors are difficult to accurately predict before surgery, and therefore, it is inappropriate to select them as factors predicting whether a RBC transfusion will be performed before surgery. There are also several reports of adjunctive surgical procedure, and the experience of the surgeon is not predictor for RBC transfusion 2, 28. In the present study, the frequency of additional surgical procedures (genioplasty, tooth extraction, and mandibular angle reduction) were also different between the two groups, with a significant difference in univariate analysis. Multivariate analysis revealed that EBL and preoperative Hb were the factors that predicting whether RBC transfusion was performed during BOS, but preoperative Hb was the only factor that could be accurately evaluated before surgery. This study found that the preoperative Hb level was significantly lower in the group who underwent RBC transfusion. There have been reports that preoperative Hb level is a predictor of transfusion in other kinds of surgery 29, 30, but as far as we know, there have been no reports showing that it can be an independent predictor even in BOS.

It has been reported that blood loss during BOS can be reduced by using induced hypotension anesthesia 17, 31, 32. In the present study, we maintained the mean arterial pressure between 50 and 65 mmHg only during the main surgical procedure with an overall operation average of approximately 67.7 mmHg. There have been reports that blood pressure was associated with blood loss 1, 33, but in this study, mean arterial pressure did not show a significant difference between the two groups.

Other methods, besides induced hypotension anesthesia, also minimize EBL during orthognathic surgery. Several studies have shown that the intravenous administration or oral irrigation of tranexamic acid are effective in reducing EBL 24, 34, 35. Desmopressin also affects perioperative bleeding by altering the activity of the von Willebrand factor, which is used in many surgical procedures with bleeding tendencies 36. Lee et al. showed that acute normovolemic hemodilution can reduce EBL and homologous transfusion when used with PABD and induced hypotensive anesthesia 37. The head-up tilt positioning is a traditional and effective way to reduce excessive EBL by stabilizing the amount of blood flowing to the head and neck regions 38. Hydroxide carboxymaltose complexes are gaining popularity as a way to increase the Hb value 39. In the present study, we demonstrated that low preoperative Hb levels increases the risk of RBC transfusions. Therefore, several methods to reduce EBL, such as induced hypotension anesthesia, the administration of tranexamic acid, desmopressin and hydroxide carboxymaltose complex, acute normovolemic hemodilution, and head-up tilt positioning can help reduce the chance of RBC transfusions.

The present study had the following limitations. First, this study was a retrospective study with a relatively small sample size. By comparison, the number of patients belonging to the TF group was relatively smaller than those of a NTF group. The retrospective study is difficult to be blinded, well-designed, or well-controlled. However, this study only included cases in which one anesthesiologist performed anesthesia and one surgeon performed surgery to reduce the bias, and excluded patients who underwent PABD. If a multicenter study is conducted with a large number of patients in a situation where the bias is controlled, the reliability can be increased further. Second, we cannot rule out the possibility that other factors that are not included in our dada have influenced RBC transfusion. We tried to collect as much data as possible through EMR. However, data collection was limited because of the retrospective nature of this study. Third, the criteria for RBC transfusion were not uniform in our study subjects. As described in the method section, RBC transfusion was performed when the Hb value was 7-8 g/dL or less, and/or when the anesthesiologist determined that RBC transfusion was necessary. Lastly, another specific limitation is EBL. EBL was calculated by subtracting the volume of saline solution used for irrigation from the total volume accumulated in the suction unit because the amount of small bone pieces, saliva, and soaked gauze could not be precisely calculated. However, since similar types of surgery were performed by a single surgeon, the blood loss excluded from the calculation is expected to be similar in all patients.

## Conclusion

RBC transfusion is an essential procedure to save a patient's life and increase long-term success rate of the surgery, but it is expensive and can result in several complications. The chance of RBC transfusions during surgery should be reduced, if possible, by taking steps to reduce the bleeding. This study shows that preoperative Hb is an important predictor for RBC transfusion in BOS. Therefore, if a patient who will undergo BOS has a low preoperative Hb, surgical and anesthetic considerations are required to reduce EBL during surgery.

## Supplementary Material

Supplementary table S1.

## Figures and Tables

**Table 1:** Demographic characteristics and past history of patients who underwent bimaxillary orthognathic surgery

Variables	NTF group (n = 256)	TF group (n = 49)	*P*-Value
**Demographic data**			
Age (years)	23.4 ± 5.2	21.8 ± 4.4	0.042
Gender (male/female)	127/129 (49.6/50.4)	18/31 (36.7/63.3)	0.119
Body weight (kg)	65.2 ± 14.1	56.8 ± 11.3	< 0.001
Height (cm)	169.4 ± 8.9	164.1 ± 8.2	< 0.001
BMI (kg/m^2^)	22.6 ± 3.9	21.0 ± 3.2	0.007
**Past medical history**			
Cardiovascular disease	2 (0.8)	1 (2)	0.410
Pulmonologic disease	5 (2)	0 (0)	1.000
Hematologic disease	3 (1.2)	1 (2)	0.506
Endocrine disease	3 (1.2)	0 (0)	1.000
Hepatologic disease	1 (0.4)	0 (0)	1.000
Psychologic disease	13 (1.2)	1 (2)	0.506
Genetic disease	1 (0.4)	1 (2)	0.296
Neurologic disease	1 (0.4)	0 (0)	1.000
Allergy	29 (11.3)	7 (14.3)	0.628
Others	12 (4.7)	1 (2)	0.701
**Past surgical history**			
Orthognathic surgery	7 (2.7)	1 (2)	1.000
Surgery related with facial cleft	15 (5.9)	2 (4.1)	1.000
Orofacial surgery	38 (14.8)	6 (12.2)	0.825
Chest surgery	5 (2)	1 (2)	1.000
Abdomen surgery	11 (4.3)	3 (6.1)	0.478
Limb surgery	5 (2)	1 (2)	1.000
Spine surgery	2 (0.8)	1 (2)	0.410
Others	13 (5.1)	4 (8.2)	0.492
**ASA Classification**			
Class I	247 (96.5)	46 (93.9)	0.417
Class II	9 (3.5)	3 (6.1)	

The values are expressed as the mean ± standard variation or number (%). ASA: American Society of Anesthesiologists; BMI: body mass index; NTF: patients who were not transfused with red blood cells; others of past medical history: otitis media, strabismus, scoliosis, and skin burn; TF: patients who were transfused with red blood cells.

**Table 2:** Preoperative variables of the patients who underwent bimaxillary orthognathic surgery

Variables	NTF group (n = 256)	TF group (n =49)	*P* -Value
**ABO type**			
A	81 (31.6)	13 (26.5)	0.247
B	67 (26.2)	9 (18.4)	
AB	36 (14.1)	12 (24.5)	
O	72 (28.1)	15 (30.6)	
HBsAg	1 (0.4)	0 (0)	1.000
Anti-HCV B	1 (0.4)	1 (2)	0.296
Anti-HBs B	95 (37.1)	22 (44.9)	0.337
**Preoperative condition**			
Systolic blood pressure (mmHg)	119.5 ± 14.6	114.9 ± 16.5	0.051
Diastolic blood pressure (mmHg)	78.3 ± 9.5	75.4 ± 11.5	0.058
Heart rate (beats/min)	80.7 ± 12.2	82.4 ± 12.9	0.374
Body temperature (°C)	36.7 ± 0.3	36.9 ± 0.4	0.011
Hemoglobin (g/dL)	14.7 ± 1.4	12.9 ± 1.8	< 0.001
Hematocrit (%)	43.4 ± 3.7	38.4 ± 5.1	< 0.001
Platelet count (×10^9^/L)	259.8 ± 53.2	250.6 ± 53.5	0.266
**aPTT (seconds)**	38.0 ± 5.0	38.6 ± 3.6	0.426
INR	1.02 ± 0.06	1.04 ± 0.06	0.004
PT (seconds)	98.0 ± 9.0	94.0 ± 9.0	0.004
Calcium (mg/dL)	9.4 ± 0.3	9.3 ± 0.4	0.654
Phosphorus (mg/dL)	3.7 ± 0.4	3.7 ± 0.4	0.865
Glucose (mg/dL)	89.0 ± 7.8	87.5 ± 7.3	0.209
**Blood urea nitrogen (mg/dL)**	11.6 ± 3.3	11.4 ± 3.5	0.592
Uric acid (mg/dL)	5.4 ± 1.5	5.1 ± 1.4	0.119
Cholesterol (mg/dL)	180.1 ± 30.7	176.4 ± 31.9	0.446
Total protein (g/dL)	7.6 ± 0.4	7.6 ± 0.4	0.998
Albumin (g/dL)	4.6 ± 0.3	4.6 ± 0.2	0.985
Total bilirubin (mg/dL)	0.8 ± 0.4	0.8 ± 0.3	0.713
Alkaline phosphatase (IU/L)	64.4 ± 17.4	63.3 ± 13.5	0.671
Aspartate transaminase (IU/L)	21.3 ± 8.9	20.5 ± 5.6	0.518
Alanine transaminase (IU/L)	21.5 ± 20.8	17.3 ± 10.5	0.171
Creatinine (mg/dL)	0.9 ± 0.2	0.8 ± 0.2	0.050
Sodium (mEq/L)	141.4 ± 1.5	141.4 ± 1.6	0.856
Potassium (mEq/L)	4.3 ± 0.3	4.3 ± 0.3	0.722
Chloride (mEq/L)	103.7 ± 1.7	104.0 ± 1.9	0.317

The values are expressed as the mean ± standard variation or number (%). Anti-HBs B: hepatitis B virus surface antibody blood test; Anti-HCV B: hepatitis C virus antibody blood test; aPTT: activated partial thromboplastin time; HBsAg: hepatitis B virus surface antigen; INR: international normalized ratio; NTF: patients who were not transfused with red blood cells; PT: prothrombin time; TF: patients who were transfused with red blood cells.

**Table 3:** Intraoperative surgical and anesthetic variables of patients who underwent bimaxillary orthognathic surgery

Variables	NTF group (n = 256)	TF group (n = 49)	*P*-Value
**Adjunctive surgical procedures**			
Genioplasty	106 (41.4)	31 (63.3)	0.007
Segmental osteotomy (Maxilla)	37 (14.5)	12 (24.5)	0.090
Turbinectomy	42 (16.4)	9 (18.4)	0.834
Tooth extraction	43 (16.8)	17 (34.7)	0.006
Mandibular angle reduction	38 (14.8)	18 (36.7)	0.001
Glossectomy	5 (2)	1 (2)	1.000
Iliac bone graft	1 (0.4)	1 (2)	0.296
Face lifting (Thread)	5 (2)	1 (2)	1.000
**Technique of anesthesia**			
Total intravenous anesthesia	151 (59)	26 (53.1)	0.528
Volatile anesthesia	105 (41)	23 (46.9)	

The values are expressed as the number (%). NTF: patients who were not transfused with red blood cells; TF: patients who were transfused with red blood cells.

**Table 4:** Intraoperative anesthetic values of patients who underwent bimaxillary orthognathic surgery

Variables	NTF group (n = 256)	TF group (n = 49)	*P*-Value
**Time**			
Duration of surgery (min)	362.7 ± 90.0	448.5 ± 115.0	< 0.001
Duration of anesthesia (min)	416.3 ± 93.4	498.4 ± 120.1	< 0.001
**Anesthetic agent**			
Propofol (mg)	2217.3 ± 1941.9	2088.4 ± 2109.3	0.675
Remifentanil (mg)	8.2 ± 7.7	7.6 ± 4.1	0.586
Thiopental (mg)	73.8 ± 145.4	68.4 ± 132.6	0.807
Rocuronium (mg)	62.7 ± 24.7	63.0 ± 28.3	0.953
Crystalloid (mL)	3402.8 ± 1164.3	4513.3 ± 1518.6	< 0.001
Colloid (mL)	560.7 ± 468.2	918.4 ± 358.6	< 0.001
Total fluid (mL)	3963.5 ± 1255.5	5431.6 ± 1612.9	< 0.001
EBL (mL)	532.6 ± 204.9	775.9 ± 324.3	< 0.001
Urine output (mL)	1079.5 ± 658.0	1260.0 ± 749.8	0.087
mBIS	43.7 ± 5.6	41.1 ± 6.0	0.023
mCompliance (ml/cm H_2_O)	40.2 ± 8.7	37.9 ± 10.0	0.137
mPIP (mmHg)	16.6 ± 3.0	16.1 ± 2.8	0.381
mSpO_2_ (%)	99.0 ± 0.7	99.3 ± 0.5	0.005
mEtCO_2_ (mmHg)	32.7 ± 2.2	32.1 ± 1.9	0.101
mPulse (beats per min)	76.3 ± 11.0	81.9 ± 12.3	0.001
mTemp (°C)	36.0 ± 0.5	35.8 ± 0.6	0.086
mA-Syst (mmHg)	110.1 ± 8.1	108.0 ± 7.3	0.089
mA-Diast (mmHg)	51.1 ± 6.2	51.1 ± 5.5	0.998
mA-Mean (mmHg)	67.7 ± 6.2	67.7 ± 5.3	0.947

The values are expressed as the mean ± standard variation. The values of BIS, Compliance, and PIP were collected every 15 minutes and the values of SpO_2_, EtCO_2_, pulse, temperature, arterial systolic blood pressure, arterial diastolic blood pressure, and arterial mean blood pressure were collected every 5 minutes during surgery. EBL: estimated intraoperative blood loss; mA Diast: mean value of the arterial diastolic blood pressure; mA Mean: mean value of the arterial mean blood pressure; mA Syst: mean value of the arterial systolic blood pressure; mBIS: mean value of the bispectral index score; mCompliance: mean value of lung compliance; mEtCO_2_: mean value of the partial pressure of end tidal carbon dioxide; mPIP: mean value of the peak inspiratory pressure; mPulse: mean value of the pulse rate; mSpO_2_: mean value of the percutaneous saturation of oxygen; mTemp: mean value of the body temperature; NTF: patients who were not transfused with red blood cells; TF: patients who were transfused with red blood cells.

**Table 5:** Intraoperative arterial blood gas analysis values of patients between the non-transfusion and transfusion groups

Variables	NTF	TF	*P*-Value
**1st ABGA (NTF:TF = 256:49)**			
pH	7.5 ± 0.1	7.5 ± 0.1	0.764
pCO_2_ (mmHg)	35.8 ± 4.5	35.5 ± 5.7	0.690
pO_2_ (mmHg)	246.9 ± 63.4	286.0 ± 77.9	0.002
HCO_3_^-^ (mEq/L)	26.4 ± 2.9	25.8 ± 2.1	0.206
SpO_2_ (%)	99.9 ± 0.5	99.9 ± 0.1	0.010
Hb (g/dL)	13.1 ± 1.4	12.5 ± 1.7	0.007
Hct (%)	38.7 ± 4.2	36.9 ± 5.1	0.011
Na^+^ (mmol/L)	138.9 ± 2.7	139.2 ± 2.2	0.578
K^+^ (mmol/L)	3.5 ± 0.4	3.4 ± 0.4	0.141
Ca^+^ (mmol/L)	1.11 ± 0.05	1.09 ± 0.04	0.078
Glucose (mg/dL)	118.7 ± 24.9	118.2 ± 25.9	0.894
**2nd ABGA (NTF:TF = 254:48)**			
pH	7.5 ± 0.1	7.5 ± 0.1	0.821
pCO_2_ (mmHg)	35.2 ± 3.6	35.1 ± 3.7	0.810
pO_2_ (mmHg)	222.9 ± 44.1	245.7 ± 52.3	0.002
HCO_3_^-^ (mEq/L)	25.1 ± 2.5	24.9 ± 2.4	0.592
SpO_2_ (%)	99.9 ± 0.4	100.0 ± 0.1	0.005
Hb (g/dL)	12.0 ± 1.6	10.8 ± 1.9	< 0.001
Hct (%)	35.4 ± 4.6	32.0 ± 5.6	< 0.001
Na^+^ (mmol/L)	139.5 ± 2.6	139.8 ± 2.4	0.386
K^+^ (mmol/L)	3.6 ± 0.4	3.6 ± 0.3	0.454
Ca^+^ (mmol/L)	1.10 ± 0.06	1.07 ± 0.05	0.026
Glucose (mg/dL)	130.6 ± 20.8	136.8 ± 22.9	0.067
**3rd ABGA (NTF:TF = 225:44)**			
pH	7.5 ± 0.1	7.4 ± 0.1	0.662
pCO_2_ (mmHg)	35.7 ± 3.5	36.1 ± 4.0	0.512
pO_2_ (mmHg)	220.5 ± 42.2	245.1 ± 45.2	< 0.001
HCO_3_^-^ (mEq/L)	24.9 ± 2.4	24.7 ± 2.9	0.539
SpO_2_ (%)	99.9 ± 0.3	100.0 ± 0.1	< 0.001
Hb (g/dL)	10.9 ± 1.5	8.9 ± 1.3	< 0.001
Hct (%)	32.2 ± 4.6	26.3 ± 4.0	< 0.001
Na^+^ (mmol/L)	139.9 ± 2.7	139.3 ± 2.4	0.171
K^+^ (mmol/L)	3.7 ± 0.3	3.6 ± 0.4	0.358
Ca^+^ (mmol/L)	1.13 ± 0.66	1.04 ± 0.06	0.372
Glucose (mg/dL)	135.4 ± 17.3	139.9 ± 17.3	0.115
**4th ABGA (NTF:TF = 88:34)**			
pH	7.4 ± 0.1	7.4 ± 0.1	0.548
pCO_2_ (mmHg)	36.8 ± 3.5	35.5 ± 4.9	0.092
pO_2_ (mmHg)	212.8 ± 61.2	241.3 ± 47.3	0.016
HCO_3_^-^ (mEq/L)	24.2 ± 2.3	23.0 ± 3.3	0.063
SpO_2_ (%)	99.9 ± 0.4	99.9 ± 0.3	0.433
Hb (g/dL)	10.3 ± 1.4	8.3 ± 1.2	< 0.001
Hct (%)	30.5 ± 4.1	24.4 ± 4.0	< 0.001
Na^+^ (mmol/L)	139.2 ± 2.5	139.4 ± 2.4	0.758
K^+^ (mmol/L)	3.9 ± 0.3	3.8 ± 0.4	0.162
Ca^+^ (mmol/L)	1.10 ± 0.07	1.02 ± 0.06	< 0.001
Glucose (mg/dL)	144.9 ± 18.8	148.4 ± 21.4	0.372
**5th ABGA (NTF:TF = 23:15)**			
pH	7.4 ± 0.1	7.4 ± 0.1	0.418
pCO_2_ (mmHg)	37.7 ± 3.3	36.9 ± 7.1	0.621
pO_2_ (mmHg)	214.3 ± 86.2	286.2 ± 135.2	0.052
HCO_3_^-^ (mEq/L)	23.9 ± 2.8	22.1 ± 3.1	0.068
SpO_2_ (%)	99.8 ± 0.4	100.0 ± 0.0	0.026
Hb (g/dL)	10.6 ± 1.6	8.8 ± 1.4	0.001
Hct (%)	31.1 ± 4.6	25.9 ± 4.2	0.001
Na^+^ (mmol/L)	138.4 ± 3.2	139.2 ± 3.1	0.441
K^+^ (mmol/L)	4.1 ± 0.4	4.1 ± 0.4	0.844
Ca^+^ (mmol/L)	1.12 ± 0.05	1.05 ± 0.07	0.002
Glucose (mg/dL)	141.1 ± 14.9	150.4 ± 25.8	0.166

The values are expressed as the mean ± standard variation. The values of ABGA were collected every 2 hours during the operation. When surgery was finished, the ABGA was no longer performed. ABGA: arterial blood gas analysis; Ca: calcium; Hb: hemoglobin; HCO_3_^-^: bicarbonate; Hct: hematocrit; K: potassium; Na: sodium; NTF: patients who were not transfused with red blood cells; pCO_2_: partial pressure of carbon dioxide; pO_2_: partial pressure of oxygen; SpO_2_: arterial oxygen saturation; TF: patients who were transfused with red blood cells.

**Table 6:** Logistic regression analysis of factors independently predicting red blood cell transfusion in bimaxillary orthognathic surgery

Variables	Univariate analysis		Multivariate analysis	
	OR (95% CI)	*P*-Value	OR (95% CI)	*P*-Value
Age	0.924 (0.855-0.998)	0.044	0.928 (0.851-1.012)	0.090
BMI	0.873 (0.790-0.965)	0.008		
**Genioplasty**				
No (reference)	1.000	0.006		
Yes	2.437 (1.296-4.584)			
**Extraction**				
No (reference)	1.000	0.005		
Yes	2.632 (1.342-5.160)			
**Bony grinding**				
No (reference)	1.000	< 0.001		
Yes	3.331 (1.696-6.544)			
Preoperative Hb	0.478 (0.379-0.604)	< 0.001	0.413 (0.310-0.551)	< 0.001
Preoperative PT	0.950 (0.916-0.985)	0.005	0.957 (0.913-1.002)	0.062
Surgical time	1.008 (1.005-1.011)	< 0.001		
Total fluid	1.001 (1.000-1.001)	< 0.001		
EBL	1.004 (1.002-1.005)	< 0.001	1.005 (1.003-1.007)	< 0.001
mPulse	1.045 (1.016-0.074)	0.002		

BMI: body mass index; CI: confidence interval; EBL: estimated intraoperative blood loss; Hb: hemoglobin; mPulse: mean value of pulse rate; OR: odds ratio; PT: prothrombin time.

## Abbreviations

ABGA: arterial blood gas analysis
ASA: American Society of Anesthesiologists
BIS: bispectral
BMI: body mass index
BOS: bimaxillary orthognathic surgery
BSSO: bilateral sagittal split osteotomy
EBL: estimated intraoperative blood loss
ECG: electrocardiography
EMR: electronic medical record
Hb: hemoglobin
Hct: hematocrit
INR: international normalized ratio
LFI: Le Fort I
NTF: patients who were not transfused with red blood cells
PABD: preoperative autologous blood donation
PT: prothrombin time
RBC: red blood cell
TF: patients who were transfused with red blood cells
