# Supplementary material for: Predictors of Red Blood Cell Transfusion in Bimaxillary Orthognathic Surgery: A Retrospective Study
Source: Int J Med Sci. 2021 Jan 29;18(6):1432–41. doi: 10.7150/ijms.55567 (PMC7893559; doi:10.7150/ijms.55567)
Supplement: Supplementary file 1 — Supplementary table S1. [file ijmsv18p1432s1.pdf]

Supplementary materials

Table S1. Differences in baseline characteristics and intraoperative variables between the smaller and larger volume red blood cell transfusion groups

Variables	Smaller volume transfusion		Larger volume transfusion		*P* -Value
	n	Data	n	Data	
Age (years)	39	22.2 ± 4.7	10	20.5 ± 2.8	0.424
Gender (male/female)	14/25	35.9/64.1%	4/6	40/60.0%	1.000
Body weight (kg)	39	58.4 ± 11.6	10	50.7 ± 7.6	0.064
Height (cm)	39	164.9 ± 7.7	10	160.8 ± 9.9	0.274
BMI (kg/m^2)	39	21.3 ± 3.1	10	19.7 ± 3.5	0.092
Adjunctive surgical procedures					
Genioplasty	22	56.4%	9	90.0%	0.070
Segmental osteotomy of the maxilla	8	20.5%	4	40.0%	0.233
Turbinectomy	8	20.5%	1	10.0%	0.663
Extraction	11	28.2%	6	60.0%	0.075
Mandibular angle reduction	12	30.8%	6	60.0%	0.141
Glossectomy	0	0.0%	1	10.0%	0.204
Iliac bone graft	1	2.6%	0	0.0%	1.000
Face lifting	1	2.6%	0	0.0%	1.000
Past medical history					
Cardiovascular disease	1	2.6%	0	0.0%	1.000
Pulmonologic disease	0	0.0%	0	0.0%	
Hematologic disease	1	2.6%	0	0.0%	1.000
Endocrine disease	0	0.0%	0	0.0%	
Hepatologic disease	0	0.0%	0	0.0%	
Psychologic disease	1	2.6%	0	0.0%	1.000
Genetic disease	0	0.0%	1	10.0%	0.204
Neurologic disease	0	0.0%	0	0.0%	
Allergy	5	12.8%	2	20.0%	0.620

Others	1	2.6%	0	0.0%	1.000
Past surgical history					
Orthognathic surgery	1	2.6%	0	0.0%	1.000
Surgery related with intraoral cleft	1	2.6%	1	10.0%	0.370
Orofacial surgery	5	12.8%	1	10.0%	1.000
Chest surgery	1	2.6%	0	0.0%	1.000
Abdomen surgery	2	5.1%	1	10.0%	0.504
Limb surgery	1	2.6%	0	0.0%	1.000
Spine surgery	1	2.6%	0	0.0%	1.000
Others	4	10.3%	0	0.0%	0.569
ASA Classification					
Class I	37	94.9%	9	90.0%	0.504
Class II	2	5.1%	1	10.0%	
ABO type					
A	9	23.1%	4	40.0%	0.736
B	7	17.9%	2	20.0%	
AB	10	25.6%	2	20.0%	
O	13	33.3%	2	20.0%	
Technique of anesthesia					
Total intravenous anesthesia	19	48.7%	7	70.0%	0.299
Gas anesthesia	20	51.3%	3	30.0%	
HBsHg	0	0.0%	0	0.0%	
Anti_HCV_B	1	2.6%	0	0.0%	1.000
Anti_HBs_B	18	46.2%	4	40.0%	1.000
Preoperative condition					
Systolic blood pressure (mmHg)	39	116.5 ± 15.6	10	108.8 ± 19.5	0.118
Diastolic blood pressure (mmHg)	39	77.0 ± 12.0	10	69.1 ± 6.3	0.087
Heart rate (beats/min)	39	83.4 ± 12.5	10	78.4 ± 14.7	0.321
Body temperature (°C)	39	36.9 ± 0.4	10	36.8 ± 0.4	0.525
Hemoglobin (g/dL)	39	13.0 ± 1.8	10	12.7 ± 1.7	0.611
Hematocrit (%)	39	38.6 ± 5.1	10	37.6 ± 5.0	0.527

Platelet count (X10⁹/L)	39	252.9 ± 54.3	10	241.5 ± 52.1	0.585
aPTT (seconds)	39	38.4 ± 3.6	10	39.4 ± 3.9	0.700
INR	39	1.0 ± 0.1	10	1.1 ± 0.1	0.196
PT (seconds)	39	95.1 ± 8.4	10	89.8 ± 10.5	0.184
Serum calcium level (mg/dL)	39	9.3 ± 0.3	10	9.2 ± 0.5	0.980
Serum phosphorus (mg/dL)	39	3.7 ± 0.4	10	3.6 ± 0.4	0.357
Blood glucose level (mg/dL)	39	87.4 ± 7.2	10	88.0 ± 7.9	0.526
Blood urea nitrogen (mg/dL)	39	11.6 ± 3.7	10	10.6 ± 2.5	0.662
Uric acid (mg/dL)	39	5.2 ± 1.5	10	4.7 ± 1.1	0.464
Cholesterol (mg/dL)	39	180.8 ± 32.6	10	159.5 ± 23.0	0.061
Total protein (g/dL)	39	7.6 ± 0.4	10	7.6 ± 0.4	0.871
Albumin (g/dL)	39	4.6 ± 0.2	10	4.5 ± 0.2	0.670
Total bilirubin (mg/dL)	39	0.8 ± 0.3	10	0.9 ± 0.4	0.293
Alkaline phosphatase (IU/L)	39	62.7 ± 13.4	10	65.4 ± 14.4	0.427
Aspartate transaminase (IU/L)	39	21.0 ± 5.8	10	18.5 ± 4.1	0.242
Alanine transaminase (IU/L)	39	18.1 ± 11.3	10	14.2 ± 5.8	0.486
Creatinine (mg/dL)	39	0.8 ± 0.2	10	0.8 ± 0.2	0.573
Sodium (mEq/L)	39	141.4 ± 1.6	10	141.6 ± 2.0	0.770
Potassium (mEq/L)	39	4.3 ± 0.3	10	4.2 ± 0.2	0.161
Chloride (mEq/L)	39	104.0 ± 1.8	10	103.8 ± 2.3	0.589
Surgical time (min)	39	436.0 ± 116.3	10	497.0 ± 100.9	0.059
Anesthetic time (min)	39	484.7 ± 121.2	10	551.5 ± 104.8	0.051
Anesthetic agent					
Propofol	39	1996.2 ± 2117.5	10	2448.0 ± 2148.4	0.650
Remifentanil	39	7.3 ± 4.3	10	8.8 ± 3.1	0.302
Thiopental	39	73.1 ± 139.0	10	50.0 ± 108.0	0.721
Muscle relaxant used					
Esmerone	39	62.6 ± 25.6	10	64.5 ± 38.6	0.950
Cisatracurium	39	0.6 ± 2.7	10	1.0 ± 3.2	0.591

Crystalloid infused (mL)	39	4379.5 ± 1348.4	10	5035.0 ± 2059.5	0.479
Volulyte infused (mL)	39	884.6 ± 388.3	10	1050.0 ± 158.1	0.119
Fluid infused (mL)	39	5264.1 ± 1484.9	10	6085.0 ± 1992.1	0.315
EBL (mL)	39	739.5 ± 288.9	10	918.0 ± 424.9	0.205
Urine output (mL)	39	1224.1 ± 768.4	10	1400.0 ± 691.9	0.385
mCompliance	29	39.3 ± 10.1	8	32.8 ± 8.3	0.190
mBIS	22	41.6 ± 5.9	7	39.4 ± 6.5	0.575
$mSpO_2$	39	99.2 ± 0.5	10	99.5 ± 0.4	0.126
$mEtCO_2$	39	32.3 ± 1.7	10	31.4 ± 2.5	0.165
mPIP	39	16.3 ± 2.7	10	15.7 ± 3.0	0.487
mPulse	39	82.1 ± 10.8	10	81.4 ± 17.5	0.766
mTemp	39	36.0 ± 0.5	10	35.3 ± 0.6	0.004
mA_Syst	39	107.7 ± 6.2	10	108.9 ± 11.2	0.941
mA_Diast	39	51.0 ± 5.3	10	51.7 ± 6.5	0.519
mA_Mean	39	67.5 ± 4.6	10	68.3 ± 7.9	0.951

The data values are expressed as the mean ± standard variation or percentage. Anti-HBs B: hepatitis B virus surface antibody blood test; Anti-HCV B: hepatitis C virus antibody blood test; aPTT: activated partial thromboplastin time; ASA: American Society of Anesthesiologists; BMI: body mass index; EBL: estimated intraoperative blood loss; HBsAG: hepatitis B virus surface antigen; INR: international normalized ratio; mA Diast: mean value of the arterial diastolic blood pressure; mA Mean: mean value of the arterial mean blood pressure; mA Syst: mean value of the arterial systolic blood pressure; $mEtCO_2$: mean value of the partial pressure of end tidal carbon dioxide; mPIP: mean value of the peak inspiratory pressure; mPulse: mean value of the pulse rate; mSpO2: mean value of the percutaneous saturation of oxygen; mTemp: mean value of the body temperature; others of past medical history: otitis media, strabismus, scoliosis, and skin burn; PT: prothrombin time.
